# Estimation formula of finished bridge pre-camber in continuous rigid-frame bridges

**DOI:** 10.1038/s41598-022-20449-4

**Published:** 2022-09-26

**Authors:** Sisi Yao, Biao Peng, Luyao Wang, Hengda Chen

**Affiliations:** 1 Key Laboratory of Degraded and Unused Land Consolidation Engineering, Ministry of Natural Resources, Xi’an, 710075 China; 2 Institute of Land Engineering and Technology, Shaanxi Provincial Land Engineering Construction Group Co., Ltd, Xi’an, 710075 China; 3 Shaanxi Provincial Key Laboratory of Bridges and Tunnels, Chang’an University, Xi’an, 710064 China; 4 School of Human Settlements and Civil Engineering, Xi’an Jiaotong University, Xi’an, 710049 China

**Keywords:** Engineering, Mathematics and computing

## Abstract

Continuous rigid-frame bridges are widely used, but the large deflection in the mid-span during operation has always been their disease. This problem is generally solved by setting the finished bridge pre-camber. There are many calculation methods for pre-camber, and the effects are different. In this paper, based on a large number of design parameters of continuous rigid-frame bridges obtained from the investigation, 18 finite element analysis models of different span combinations were established, and 30 sets of valid data were obtained under the action of multi-factor. The results show that the shrinkage and creep of concrete is the most important factor for the mid-span deflection of continuous rigid frame bridges, and the deflection amount has an obvious functional relationship with the span. The effect of prestress loss on mid-span deflection is second, and stiffness reduction has little effect on mid-span long-term deflection. In this paper, the least-squares method is used to perform polynomial fitting, and the fitting formula for the mid-span finished bridge pre-camber is finally obtained. The applicability of the calculation formula is proved by comparing it with the specification solution, the empirical solution, and the measured value.

## Introduction

**Figure 1:**
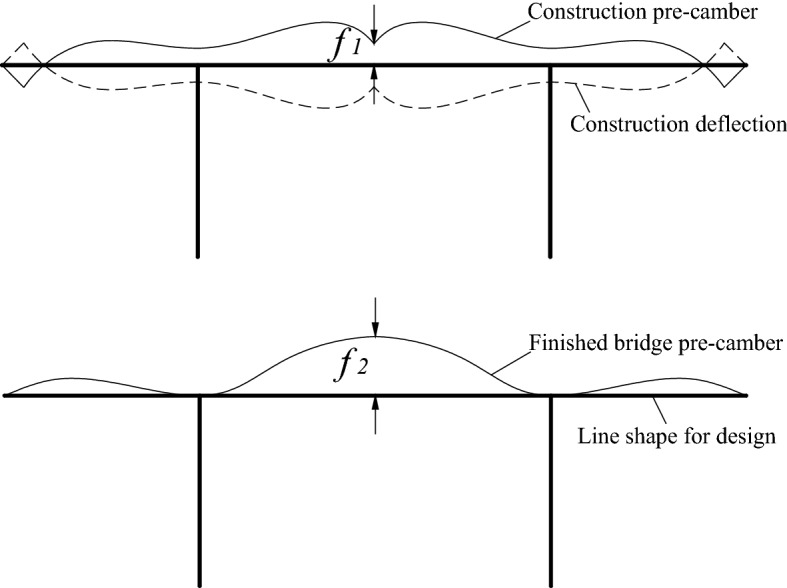
Pre-camber of continuous rigid frame bridges.

The continuous rigid-frame bridge constructed by the cantilever pouring method needs to set the construction pre-camber *f*_1_ and the finished bridge pre-camber *f*_2_ (Fig. 1). The value of *f*_2_ is determined according to the mid-span deflection value during the operation period. To ensure the smoothness of the line shape of the continuous rigid frame bridge during the operation period, the pre-camber value *f*_2_ is often set to offset the excessive mid-span deflection during the operation period. However, the selection of the pre-camber value is related to various factors such as the structural span, material, etc. For long-span continuous rigid-frame bridges, the single-bridge modeling analysis is often performed in the design stage to obtain the numerical solution of *f*_2_. In the case of not calculating the single bridge model, the designer often selects the local experience value, and there is no unified standard. In China's Specification for Design of Highway Reinforced Concrete and Prestressed Concrete Bridges and Culverts (JTG 3362-2018)^1^, the deflection growth coefficient $\eta \theta ,Ms$ is used to calculate the long-term deflection of the structure. The deflection growth coefficient is related to the concrete material of the main beam, and its value is actually an empirical value. However, the calculated value is quite different from the actual deflection value of the bridge, which cannot make the final linear shape of the structure smooth^2–5^.

Many scholars have studied the factors that affect the excessive mid-span deflection of continuous rigid-frame bridges during operation and proposed that the bridge deflection monitoring should be tracked in the later period^6–8^. But they could not propose a reliable method for calculating the finished bridge mid-span pre-camber value. Some bridge designers conducted research on reasonable pre-camber for a single bridge, but they are not universal^9–11^. Yang Z.P. tracked and measured the mid-span deflection of a continuous rigid frame bridge with a main span of 270 m and obtained relevant data^12^. Wang P.J. analyzed these measured data and corrected the long-term growth coefficient, but the conclusions drawn from only one bridge data are difficult to apply to bridges of various spans^13^. Based on these measured data, He S.H. proposed the deflection growth coefficient correction coefficient *K* and predicted the change value of the mid-span deflection through the *K* value fitting curve^14^. However, to obtain the *K* value, a corresponding calculation model needs to be established.

Although there have been many studies on the influencing factors of the mid-span deflection of continuous rigid-frame bridges, a method to correct the deflection growth coefficient is proposed based on a small amount of measured data. However, due to a lack of data, the value of the finished bridge pre-camber of the rigid frame bridge is still based on qualitative understanding, and the quantitative calculation formula is basically in the blind area. The value of *f*_*2*_ of continuous rigid-frame bridges in various places still adopts the empirical formula:

1 $$f_{2} = l/1000 - d/2$$

where *l* is the main span of the bridge, and *d* is the mid-span deflection under live load. With the improvement of the main beam concrete label in recent years, there have been many cases in which the selection of empirical values is too conservative, resulting in obvious mid-span upper arches of continuous rigid-frame bridges during operation. It affects the driving comfort. Design units reduced this value from 1/1000 to 1/1500, but the empirical value lacks a calculation basis and cannot be generally applied to continuous rigid-frame bridges of different spans. Based on a large number of real bridge survey data, this paper establishes continuous rigid-frame bridge models with different spans and performs finite element calculations under the influence of multi-factors one by one. By analyzing the calculation results, a method for selecting the finished bridge pre-camber value with wide applicability is obtained. After comparative analysis, its reliability is verified, which provides a reference for setting the finished bridge pre-camber value of continuous rigid-frame bridges.

## Reasonable value of influencing factors

According to previous research results, it is clear that the shrinkage and creep of concrete, the reduction of structural stiffness, and the loss of longitudinal prestress are the three main factors that affect the mid-span deflection of continuous rigid frame bridges during the operation period^12–16^. To further analyze the influence of the coupling effect of each factor on the structural deflection, it is particularly important to select a reasonable value.

### Shrinkage and creep of concrete

The creep of concrete often leads to an increase in the deflection of the beam, causing a loss of prestressing, which can lead to cracking. In the early years, due to the lack of understanding of shrinkage and creep and the limitations of the regulations at that time, many bridges at home and abroad suffered different degrees of disease due to shrinkage and creep^17,18^. For example, the Parrots Ferry continuous rigid frame bridge completed in 1978 in the United States has a main span of 195 m. When tested in 1990, its mid-span deflection was as large as 635 mm; the auxiliary channel bridge of the Humen Bridge built in China in 1997 has a main span of 270 m, and the maximum deflection of mid-span was 222 mm when it was tested in 2003.

After the shrinkage and creep of the concrete continue for 6 months, the structural deformation can reach 70% ~ 80% of the final creep deformation. Then the deformation growth gradually slows down^17–20^. According to this characteristic, the calculation time of shrinkage and creep is usually set as 1000–1500 days in the design stage. Therefore, this paper takes the deflection value after 3 years as the calculation target value of this factor.

### Reduction of structural stiffness

During the operation of the structure, due to shrinkage and creep, the deformation increases, the effective prestress decreases, and cracks begin to develop continuously. Therefore, the stiffness needs to be reasonably reduced to meet the actual situation.

According to relevant research and the concrete fatigue stiffness attenuation test law^21,22^, this paper reduces the concrete stiffness of the main beam by 10% after 3 years of operation.

### Loss of longitudinal prestress

**Figure 2:**
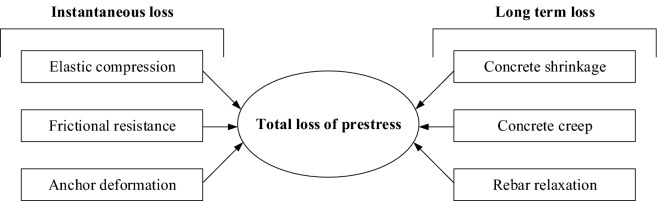
Composition of prestress loss.

The effective prestressing of steel tendons in prestressed concrete bridges will have a certain loss during construction and operation. Many factors cause the loss of prestress (Fig. 2), and some factors affect and depend on each other, it is very complicated work to accurately calculate and determine the effective prestress.

The prestressing of continuous rigid-frame bridges generally adopts the post-tensioning method. As early as 1958, the American Concrete Institute and Civil Science (ACI-ASCE) proposed the "Recommendations for the Design of Prestressed Concrete Structures", which stipulated the total loss value caused by the elastic compression, shrinkage and creep of concrete, and the relaxation of steel bars: post-tensioned members were taken as 172 MPa. With the development of engineering practice, considering the underestimation of the relaxation stress loss, the American ACI code and the American Highway Bridge Code (AASHTO) revised this in 1975, and the total loss value of the post-tensioned steel strand was taken as 228 MPa. The American Post-tensioned Concrete Institute (PTI) also revised the total prestress loss value, and the total prestress loss of the 1860 MPa steel strand was taken as 240 MPa (12.9%). Chinese-American Lin Tongyan proposed that the average total loss is 20% of the tension control stress. China has also made some statistical analysis on the total prestress loss value based on a lot of engineering practice experience, and proposed that when designing prestressed concrete structures, the effective prestress can be taken as 60%-80% of the tension control stress.

In addition, prestressed concrete continuous rigid frame bridges generally have longitudinal, vertical, and lateral prestressing in the box girder. In the modeling process of this paper, only the longitudinal prestressing is considered, and the lateral and vertical prestressing effects are ignored. In this paper, Combined with the experimental data of the indoor scale model and the measured data of the actual bridge, as well as the prediction method of the prestress loss in recent years, this paper conservatively reduces the longitudinal prestress to 70% of the control stress after 3 years of operation^23–27^.

## Modeling methods

### Structural parameters

The finite element calculation and analysis of mid-span deflection of continuous rigid-frame bridges during operation is the focus of this paper. Relying on the science and technology project of the Department of Transportation of Shaanxi Province, China, this paper conducts a large number of investigations on the continuous rigid frame bridges in service. During the investigation, bridge design parameters for different span combinations were obtained.

To obtain the mid-span deflection value of continuous rigid frame bridges with various span combinations, this paper selects a total of 18 bridges with main spans ranging from 80 to 200 m according to the design parameters obtained from the investigation.

**Figure 3:**
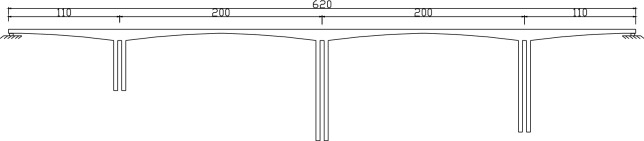
Elevation of Xushuihe bridge (*m*).

Due to limited space, the Xushuihe Bridge is selected here for parameter description (Fig. 3). Xushuihe Bridge is a 110 + 2 × 200 + 110 m prestressed concrete continuous rigid frame bridge. The main beam is a variable section box beam, and the concrete grade is C50. The pier material is C40 concrete. The bridge is constructed by the cantilever casting method. The prestressing of the box girder is constructed by the post-tensioning method. The standard value of tensile strength of prestressed steel is 1670 MPa. The highest pier is 98 m, and the construction is carried out in sections of 3 m. The construction of the bridge started in September 2002 and was completed in November 2005. It has been in operation for nearly 17 years so far.

### Model description

**Figure 4:**
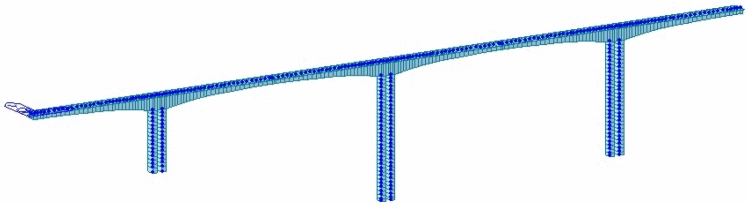
Finite element model of Xushuihe bridge.

According to the design parameters, a finite element 3D model is established by Midas civil software. The dispersion of the structure is divided according to the construction beam section of the main beam. The piers are divided into segments every 3 m. This model has a total of 363 nodes and 356 units, and they constitute the framework of the entire model (Fig. 4). The pier and main beam adopt general beam elements. Because this paper analyzes the state of the bridge after completion, the nonlinear effect of the material is ignored.

The shrinkage and creep of concrete varies with time. In the program, the shrinkage and creep of concrete are simulated by defining time-dependent material properties and automatically calculating the theoretical thickness of the element. In the process of setting the boundary conditions of the model, the lower end of the bridge pier is set as the fixed end. Use rigid connections to connect the piers to the main girder nodes to achieve pier-girder consolidation. The two ends of the prestressed box girder are set as rolling bearings along the direction of the bridge to meet the deformation of the structure under temperature changes.

### Calculation condition

According to the parameter values selected above, make corresponding modifications in the finite element model. A total of 5 calculation conditions are in this paper. In this way, the degree of influence of each factor and the degree of influence of multi-factor coupling on deflection can be solved. See Table 1 for details.

**Table 1:** List of calculation conditions.

Number	Calculate Content	Symbol	Target value
1	Deflection value at the completion of bridge construction	y_0_	–
2	Deflection value of the bridge after the stiffness is reduced by 10%	y_E_	V_E_ = y_E_ – y_0_
3	Deflection value of the bridge after the effective stress of the prestress is reduced by 30%	y_p_	V_p_ = y_p_ – y_0_
4	Considering shrinkage and creep, deflection value after 3 years of operation	y_c_	V_c_ = y_c_ – y_0_
5	Deflection value under the coupling action of three factors	y_m_	V_m_ = y_m_ – y_0_

## Results and discussion

### Finite element calculation

A total of 18 models of continuous rigid-frame bridges with different span combinations are established in this paper, and 30 sets of valid data are obtained through finite element calculation and analysis. The calculation results are shown in Table 2. In Table 2, the positive value represents the upper arch of the main beam, and the negative value represents the lower deflection of the main beam.

**Table 2:** Finite element calculation results (mm).

Number	Bridge name	Span combination (m)	Main beam concrete	Tensile strength of prestressed steel (MPa)	V_E_	V_p_	V_c_	V_m_	Live load deflection, *d*
1	Hanguguan Bridge	45 + 80 + 45	C55	1860	− 0.10	− 11.69	− 2.93	− 25.18	− 14.97
2	Yangjiabian Bridge	56 + 90 + 56	C50	1860	0.67	− 7.65	0.99	− 18.87	− 27.83
3	Qingcheng River Bridge	57 + 100 + 57	C50	1860	2.80	− 12.50	1.70	− 17.04	− 8.20
4	Qiyuan Yellow River Bridge	62.5 + 4 × 110 + 62.5	C50	1860	0.00	− 12.01	− 9.69	− 33.07	− 18.74
5					0.00	− 9.53	− 7.35	− 25.27	− 19.95
6	Juhe Bridge	62.5 + 4 × 115 + 62.5	C55	1860	0.00	− 30.00	− 12.68	− 61.71	− 27.97
7					0.00	− 19.48	− 18.68	− 50.80	− 26.49
8	Hongqi Village Yellow River Bridge	75 + 2 × 120 + 75	C55	1860	0.23	− 24.91	− 10.67	− 46.80	− 48.99
9					0.25	− 24.88	− 10.44	− 46.46	− 49.20
10	Bridge 1	65 + 6 × 120 + 65	C50	1860	0.77	− 13.52	− 23.55	− 53.52	− 22.05
11					0.82	− 12.95	− 21.90	− 48.95	− 23.36
12					0.23	− 12.21	− 24.87	− 52.14	− 24.25
13	Kuye River Bridge	68 + 4 × 130 + 68	C55	1860	2.17	− 15.40	− 10.33	− 34.39	− 49.25
14					0.80	− 18.61	− 10.19	− 36.80	− 42.38
15	Han River Bridge	75 + 140 + 75	C50	1860	0.00	− 12.51	1.88	− 32.11	− 21.57
16	Yijiahe Bridge	75 + 140 + 75	C50	1860	3.86	− 24.64	− 2.04	− 54.40	− 33.38
17	Biandangou Bridge	75 + 3 × 140 + 75	C55	1860	0.00	− 27.22	− 9.49	− 56.56	− 38.55
18					0.24	− 15.45	− 14.45	− 40.33	− 30.11
19	Nujiang Bridge	88 + 160 + 88	C55	1860	− 0.32	− 11.53	− 55.99	− 82.85	− 22.59
20	Bridge 3	85 + 3 × 160 + 85	C50	1860	1.49	− 20.95	− 19.21	− 66.94	− 29.22
21					1.36	− 16.36	− 14.02	− 54.98	− 30.09
22	Wulipo Bridge	85 + 4 × 160 + 85	C50	1860	0.00	− 15.60	− 16.24	− 62.68	− 42.04
23					0.00	− 15.74	− 16.68	− 52.87	− 44.13
24	Ziyang Han River Bridge	95 + 2 × 170 + 95	C50	1860	− 2.32	− 18.59	− 36.34	− 87.75	− 57.36
25					− 1.84	− 18.66	− 34.38	− 85.62	− 57.88
26	Bridge 2	95 + 4 × 180 + 95	C50	1860	1.46	− 28.57	− 59.32	− 103.96	− 37.90
27					2.15	− 27.63	− 56.61	− 99.76	− 46.40
28	Duifang River Bridge	100 + 180 + 100	C55	1860	− 0.62	− 14.56	− 58.68	− 90.80	− 26.06
29	Xushuihe Bridge	110 + 2 × 200 + 110	C50	1670	0.00	− 12.29	− 58.36	− 98.54	− 35.01
30					0.00	− 10.64	− 61.34	− 101.71	− 37.76

**Figure 5:**
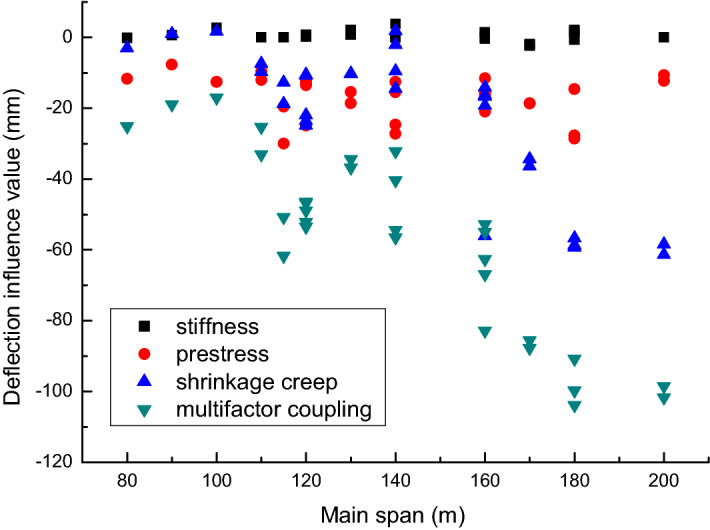
Influence of various factors on mid-span deflection.

After sorting and analyzing the solved data, the influence degree of each factor on the mid-span deflection is obtained. The scatter plot is shown in Fig. 5.

The calculation results show that: (1) The structural cracking causes the stiffness to decrease. However, the effect of stiffness reduction on mid-span deflection is only about 3 mm. The results are weakly correlated with span. It can be seen that its influence on the pre-camber value of the completed bridge can be ignored. (2) The mid-span deflection caused by the loss of longitudinal prestress is concentrated between [− 10, − 30] mm. Its value is approximately 35% of the deflection after 3 years of operation, but there is no obvious functional relationship with the span. (3) The mid-span deflection caused by the shrinkage and creep of concrete for 3 years increases with the increase of the span. And there is an obvious functional relationship. (4) The relationship between the mid-span deflection and span caused by shrinkage and creep is similar to the relationship between the mid-span deflection and span under the influence of multi-factor coupling. This further verifies the theory that concrete shrinkage and creep is the most important factor affecting the finished bridge pre-camber value of continuous rigid-frame bridges.

### Fitting pre-camber calculation formula

Through the above analysis, we know that the mid-span deflection value under the action of multi-factor coupling has an obvious functional relationship with the span. Therefore, it is possible to fit discrete data using the least-squares method.

Use the least-squares method to establish an approximate continuous model for a large number of discrete data.

Given discrete data: $(xi,f(xi))$ i = 0, 1, 2…n.

Where $xi \in \left[ {a,b} \right]$, find a function $S(x)$ as an approximate continuous model of $f$. The error between $S(xi)$ and $f\left( {xi} \right)$ is $\delta i$,

2 $$\delta_{i} = S(x_{i} ) - f(x_{i} )\quad {\text{i}} = 0,\,1,\,\,2 \ldots {\text{n}}$$

Marked as $${{\varvec{\updelta}}} = (\delta 0,\delta 1,\delta 2, \cdots \delta m)^{T}$$ i = 0,1,2…n.

Minimize the sum of squares, then

3 $$\left\| {{\varvec{\updelta}}} \right\|_{2}^{2} = \sum\limits_{i = 0}^{n} {\delta_{i}^{2} } = \min \sum\limits_{i = 0}^{n} {\left[ {S\left( {xi} \right) - f\left( {xi} \right)} \right]^{2} }$$

From this, a fitting curve that meets the requirements can be obtained.

According to the above solution ideas, polynomial fitting is performed on the span x (*m*) and the mid-span deflection value y (*mm*) under the action of multi-factor coupling.

4 $$y = f\left( {x,\overrightarrow {\beta } } \right) = \beta_{0} + \beta_{1} x + \beta_{2} x^{2}$$

Using the least-squares method to calculate the value of each undetermined coefficient. The fitted polynomial formula is

5 $$y = - 14.066 + 0.138x - 0.003x^{2}$$

The correlation coefficient of the fitted curve is 0.801. It shows that the fitted polynomial curve is well correlated with the calculated discrete data. From this, it can be obtained that the pre-camber setting value of the bridge is

6 $$f_{2} = - y - d/2$$

**Figure 6:**
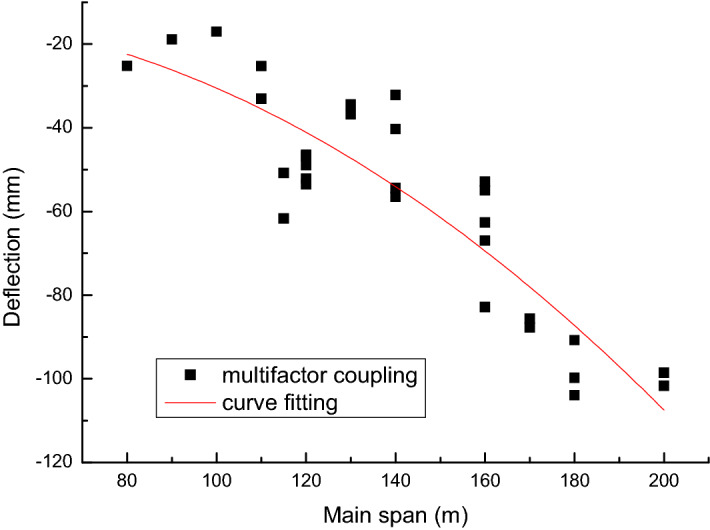
Fitting polynomial curve of the bridge deflection.

**Figure 7:**
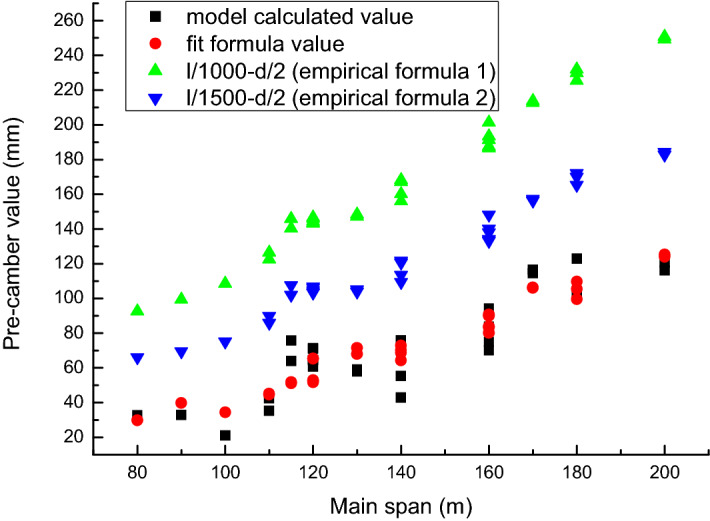
Comparison of model calculation value, fitting formula value and empirical formula value.

The fitted polynomial curve is shown in Fig. 6. The comparison of model calculation results, fitting formula results, and empirical formula results is shown in Fig. 7.

It can be seen from Fig. 7 that the fitting formula is similar to the model calculation result. The finished bridge pre-camber set by empirical formula (1) is relatively large, even if it is later revised to empirical formula (2), it is still much larger than the model calculation result. This also explains the phenomenon that the bridge is obviously arched up during the operation stage when the pre-camber is set by the empirical formula.

### Discussion

In order to further verify the applicability of the fitting calculation formula, this paper compares and discusses the current design specification solution, empirical formula solution, fitting formula solution and measured value of the pre-camber value of the completed bridge.

It is necessary to verify whether the estimation formula is reasonable through measured data. Among the 18 continuous rigid-frame bridges analyzed above, due to the long operation time of some bridges and the changes in the management and maintenance units of some bridges, complete deflection observations cannot be obtained during the investigation process. Fortunately, we found the exact mid-span elevation values for 4 of the bridges when they were built, and measured the current mid-span elevation values. The difference is used as the measured data for the comparative analysis.

According to the design specification of China (JTG 3362-2018), the finished bridge pre-camber value *f*_2_ is

7 $$f_{2} = \eta_{\theta ,Ms} \omega_{Ms} - \eta_{\theta ,pe} \delta_{pe}$$

in the above formula: $\omega_{Ms}$—Deflection value due to bending moment value calculated from the combination of short-term effects of acting (or load); $\delta_{pe}$—Camber value produced by the permanent prestress $N_{pe}$; $\eta_{\theta ,pe}$—Long-term effect growth factor, take $\eta_{\theta ,pe} = 2$; $\eta_{\theta ,\,\,Ms}$—Deflection growth factor for short-term load effect combinations considering long-term effects.

**Figure 8:**
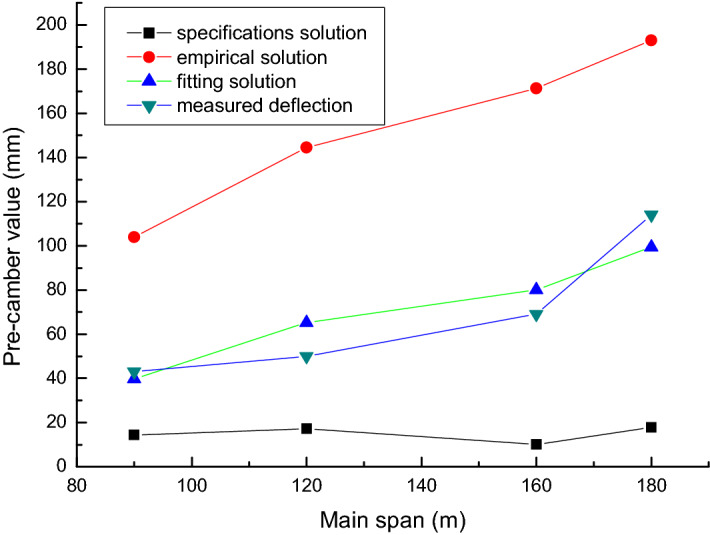
Comparison of mid-span pre-camber.

This paper discusses 4 continuous rigid frame bridges. The current design specification solution (Eq. 7), the empirical solution (Eq. 1), and the fitting formula solution (Eq. 6) for the pre-camber of the mid-span bridge are obtained respectively and are compared with the measured deflection data (Some measured data comes from Ref.^28^). The parameters of each bridge are shown in Table 3, and the comparative calculation results are shown in Table 4 and Fig. 8.

**Table 3:** Structural parameters of real bridge (mm).

Examples	Span combination (m)	Main beam concrete	Tensile strength of prestressed steel (MPa)	$\eta_{\theta ,\,\,Ms}$	$\omega_{Ms}$	$\delta_{pe}$	*d*
1	56 + 90 + 56	C50	1860	1.43	27.41	12.40	− 27.83
2	75 + 2 × 120 + 75	C55	1860	1.41	78.95	47.03	− 48.99
3	88 + 160 + 88	C55	1860	1.41	50.59	30.65	− 22.59
4	100 + 180 + 100	C55	1860	1.41	65.34	37.15	− 26.06

**Table 4:** Comparison of mid-span pre-camber (mm).

Examples	Specification solution (7)	Empirical solution (1)	Fitting solution (6)	Measured deflection	Deviation 1 (%)	Deviation 2 (%)	Deviation 3 (%)
1	14.40	103.92	39.86	43	0.33	2.42	0.93
2	17.26	144.50	65.20	50	0.35	2.89	1.30
3	10.03	171.30	80.08	69	0.15	2.48	1.16
4	17.83	193.03	99.46	114	0.16	1.69	0.87

Deviation 1 = specification solution/measured deflection × 100%; deviation 2 = empirical solution/measured deflection × 100%; deviation 3 = fitting solution / measured deflection × 100%.

The calculation results show that: (1) The pre-camber value of the completed bridge calculated according to the specification is far less than the measured deflection value. The result is difficult to meet the requirements of making the final linear shape of the continuous rigid-frame bridge smooth. (2) The pre-camber value of the completed bridge calculated according to the empirical formula is too conservative, which is about twice as large as the measured value. The bridge is still arched in the middle of the span after many years of operation, which affects the driving comfort. (3) The pre-camber value of the completed bridge calculated according to the fitting formula is the closest to the measured deflection value, which is about 0.8–1.3 times as large as the measured value. This shows that after the bridge has been in operation for 3 years, the mid-span alignment is close to the design alignment. The bridge deck is smooth and there is little change in the later period.

Therefore, the pre-camber value of the completed bridge calculated by the fitting formula in this paper has good applicability. After *f*_*2*_ is determined, according to the usual practice, the finished bridge pre-camber of the side span is 1/4 of *f*_*2*_, and it is set at 3* l*/8 of the side span. The rest of the points can be assigned according to the cosine curve.

## Conclusions and recommendations

Excessive deflection at mid-span during operation is the most common problem of continuous rigid-frame bridges. To solve this problem, by referring to the relevant research and literature, this paper selects reasonable values for the shrinkage and creep of concrete, the reduction of structural stiffness, and the loss of prestress. Based on a large number of design parameters of continuous rigid-frame bridges obtained from the investigation, 18 finite element analysis models of different span combinations were established, and 30 sets of valid data were obtained by reasonably modifying the calculation parameters. Using the least squares method to fit the discrete data, the pre-camber estimation formula of the completed bridge is obtained, and the applicability of the formula is verified. The main conclusions are as followings:

1. The stiffness reduction has little effect on the long-term deflection of the continuous rigid frame bridge. The mid-span deflection caused by the loss of prestressing accounts for about 35% of the total deflection. The shrinkage and creep of concrete is the main reason for the mid-span deflection of continuous rigid-frame bridges during operation, and the amount of deflection has an obvious functional relationship with the span.
2. Under the action of multi-factor coupling, the least-square method is used to perform polynomial fitting on a large number of model calculation data. The calculated results of the fitting formula are in good agreement with the measured deflection values of the bridge.
3. The method for selecting the finished bridge pre-camber value of continuous rigid-frame bridges proposed in this paper solves the problem that the empirical formulas are not uniform and the value is too large. This is of great significance for achieving the smoothness of the bridge deck during the operation period.

Many factors cause the mid-span deflection of continuous rigid-frame bridges during operation, and some factors influence and depend on each other. Therefore, it is very complicated work to accurately calculate the deflection value. Establishing the functional relationship between the finished bridge pre-camber and the parameters such as span, structural material, and construction method is worth further research in the future.

## Data Availability

The datasets used and analysed during the current study available from the corresponding author on reasonable request.
